# The event-driven nature of online political hostility: How offline political events make online interactions more hostile

**DOI:** 10.1093/pnasnexus/pgad382

**Published:** 2023-11-10

**Authors:** Stig Hebbelstrup Rye Rasmussen, Michael Bang Petersen

**Affiliations:** School of Business and Social Sciences, Aarhus Universitet, Aarhus 8000, Denmark; School of Business and Social Sciences, Aarhus Universitet, Aarhus 8000, Denmark

## Abstract

Hostile interactions permeate political debates on social media, but what is driving the long-term developments in online political hostility? Prior research focuses on individual-level factors such as the dispositions of users or network-level factors such as echo chambers. Moving beyond these accounts, we develop and test an event-oriented explanation and demonstrate that over the course of the 2020 election year in the United States, all major shifts in political hostility on the social media platform Twitter were driven by external offline events. Importantly, these events were magnified by Twitter users within the most politically hostile and most ideologically homogeneous networks. Further contributing to the individual and network-oriented accounts, we show that divisive offline events mobilized individual users not already disposed for hostility and may have helped facilitate the formation of echo chambers. The dynamics of online interactions—including their level of hostility—seem crucially dependent on developments in the offline world.

**Significance Statement**

We monitored the level of political hostility on American Twitter over a year of high turmoil including the onset of the COVID-19 pandemic, the murder of George Floyd, the electoral loss of Donald Trump in 2020, and the storming of the US Congress in 2021. We observe that the dynamics of hostile social media discussions about politics is closely tied to these developments in the offline world such that divisive events increase online political hostility. For authorities and practitioners working to deescalate online discussions, this provides insights that hostility is not constant and that it is a key to precisely time interventions against hostility and employ them in the immediate aftermath of divisive events.

## Introduction

While social media was initially believed to hold promise as sites for constructive deliberation regarding difficult political issues and events, the last decade has seen a sharp increase in public concerns regarding the hostile nature of political online discussions (1). The hostile nature of online political discussions has been widely documented (2, 3) and have been found to depress political engagement (1, 4) and fuel outrage and incite harassment of public figures (5–7).

Online political hostility can be defined as the expression of degrading, malicious, and intimidating online behavior toward individuals or collectives based on political differences (8). To solve the problem of hostility in online political discussions, it is key to have an accurate, evidence-based diagnose of the nature of the problem, and increasing amounts of research is dedicated to this end (9, 10). At present, two approaches dominate the research field. One approach is user-oriented and focuses on the personality of individual users, highlighting how aggressive and antisocial personality traits predispose some users to be much more aggressive than others (1, 11, 12). According to the findings from this approach, there is a high degree of stability in hostile behavior such that online hostility continuously flows from the same but often small group of individuals. The large nonhostile majority is exposed to the behavior of these hostile individuals due to the cross-cutting nature of many online interactions, further powered by algorithms (13).

The other approach is network-oriented and focuses on the role of the networks that users are embedded in. In particular, so-called echo chambers where politically like-minded persons exchange like-minded information (14, 15) are often seen as a key cause of the rapid online spread of hostile, political content (2, 3).

While some studies argue that the characterization of social media as echo chambers is exaggerated (15–17), it is also clear that online echo chambers exist (18, 19) and that they may be responsible for producing more hateful social media users through a number of distinct processes such as enclave deliberation (20, 21), motivated reasoning (22), the use of moral-emotional language (23), and norm setting (24). At the same time, recent reviews underscore how further research is needed and, in particular, how “it is important to understand the sources and mechanisms that drive echo chambers” (25).

In this article, our point of departure is a third approach for understanding the emergence of online political hostility: large-scale offline political events. We know from a wealth of studies that political attention in legacy media is heavily event-driven (26–29), and similar conclusions are beginning to emerge in studies of online activism (30–32). As such, divisive political events in the offline world may impinge and mobilize online social networks that subsequently interpret these events through polarized lenses, increasing their level of hostility. Such an event-driven model of the operations of online social networks is consistent with more general psychological models of mobilization, suggesting that so-called “trigger events” are key for the coordination of a group's attention (26, 33, 34). Political history is paved with trigger events with a significant impact on subsequent mobilization: the murder of Archduke Franz Ferdinand of Austria in 1914, triggering the First World War; the downing of the plane of Rwanda's president in 1994, which triggered the subsequent genocide; the acquittance of police officers for the beating of Rodney King in 1992, which triggered large-scale riots in Los Angeles; and the murder of George Floyd by a police officer, which triggered large-scale protests across the United States and many other countries.

Prior research has clearly shown the relevance of such offline events for discussions on social media. Some research has focused on the relationship between echo chambers and events. For example, Barberá et al. (35) found that events, such as the 2013 Boston Marathon bombing and the 2014 Super Bowl, often stimulated information exchange across ideological lines. Other research has focused on how real-world events mobilize social media users through, e.g. the widespread sharing of hashtags (30, 36) or emotions of support or rejection (31). Finally, a few studies have focused on the present research question: the event-driven dynamics of online political hostility. Siegel et al. (37), e.g. examined hate speech on Twitter during the 2016 US presidential election and found that campaign events produced spikes in hateful language but only briefly. Most prominently, Kim (27) focused on violent rhetoric on Twitter during the 2021 attack on the US Capitol and found that such rhetoric had a “close relationship with contentious offline politics.” At the same time, Kim (27) raises important avenues for future research including the need for further evidence on the involved mechanisms and the effects of extreme rhetoric. In this article, we seek to provide such evidence by examining how the impact of large-scale divisive events unfolds across users and networks.

By integrating the analysis of events with analyses of both users and networks, we provide a theoretical and empirical integration of the event-oriented approach to online political hostility with the existing user-oriented and network-oriented approaches. This integration focuses on the interaction among events, networks, and user disposition and may, we contend, involve important contributions to each approach. First, the mobilization literature suggests that divisive events may be particularly likely to activate individuals who are not already engaged (38) and, hence, events may be uniquely important instances for generating hostility among those who are not normally predisposed for hostility, according to the user-oriented approach. Second, as hostile intergroup relations may drive people toward the safety of more homogeneous networks (39), event-driven hostility may contribute to explain the drivers of echo chambers, as highlighted in the network-oriented approach.

To study the effects of offline political events on online political hostility, we take advantage of a unique data set consisting of 2,839 American Twitter users who provided informed consent for us to access and analyze their tweets. We do so over a period characterized by significant political turmoil: from the end of 2019 to the beginning of 2021. Among other events, this period includes the onset of a pandemic, the murder of George Floyd, a US presidential election, and the attack on the US Capitol. We link these individual-level data with almost 2 billion tweets from the friends and followers of these panelists to obtain behavioral measures of network characteristics [as opposed to inferred or survey-based ones e.g. 24). From this, we assess individual users’ level of Twitter activity and their tweets’ level of political hostility as well as the ideological homogeneity and hostility of the networks they are embedded within. Using these data, we zoom in on two largely exogenous and critical offline events in the United States—the murder of George Floyd and the attack on the US Capitol—and investigates what happens to individuals in social media networks when events permeate their boundaries.

## How offline political events drive online political hostility

Using longitudinal data of online political hostility at both the network and user levels, we investigate whether political hostility is driven or not by divisive offline events. From the perspective of the event-oriented approach, offline events should powerfully shape the activity of online networks and users. We allow ourselves to be exploratory in our conceptualization of what constitutes a “divisive” event. Rather than providing an a priori definition of what constitutes a “divisive events,” we opt for the more exploratory approach of simply asking whether activity and hostility in social media networks are clearly linked to ex post identifiable offline events or not.

To help organize the empirical investigation, we can formulate this as a formal empirical prediction:

- Hypothesis 1 (H1): Temporal changes in political hostility in social media networks should be clearly linked to identifiable divisive political events.

Hypothesis H1 focuses on aggregate associations between online political hostility and external events. However, this association should reflect that divisive external events exert an effect on individual behavior on social media. This is the focus of our second set of hypotheses. Political events may drive both the amount and the hostility of individual users through multiple mechanisms. Large political events may grab the attention of politically interested social media users—either directly or via the mobilization efforts of elite actors or fellow partisans—and make these users prioritize their political goals in their online interactions. This increased activity may often turn hostile, as many political events may generate a felt need for defending the in-group against accusations of a negative involvement in the event or provide an opportunity for motivated partisans to accuse the out-group of such involvement (40). These hostile interactions may intensify as activists seek to mobilize others to engage in similar behavior (41) and as, in the case of events where interpretations of what happened and why is unclear, different ideological groups fight over the correct interpretation (42). Consistent with this, prior research has shown, e.g. that divisive events such as elections can activate social media users (36) and that violent offline events are related to hostile online interactions (27, 31, 32). On this basis, our next sets of hypotheses are as follows:

- H2.1: Divisive offline events should increase the activity of social media users.
- H2.2: Divisive offline events should increase the political hostility of social media users.

Threatening situations, such as divisive political events, may either galvanize engagement among those who are already engaged or they may capture the attention and engagement of those who are not (43, 44). The literature on offline threats and political attitudes and behavior involves conflicting findings (43), and to our knowledge, no studies have examined this directly in the context of online political hostility. Yet, while prior user-oriented research has highlighted the importance of dispositions for online political hostility, some research suggests that contextual frustrations may be crucial for triggering hostility in those who are not otherwise disposed for hostility (11). Accordingly, divisive political events may be powerful accelerators of mobilization. In the empirical analyses, we explore this extension of hypothesis H2.2.

Beyond the dispositions of individual users, it is likely that the network of the users is key for understanding the dynamics of event-driven online political hostility. A central idea in the echo chamber literature is that enclave discussions and motivated reasoning can push less hostile individuals embedded in hostile and polarized networks toward radicalization (7, 21, 22, 45). In the context of divisive events, prior work leads to the expectation that more ideologically homogeneous and more hostile networks are more likely to activate in the face of adverse information and events (7, 22, 45). Furthermore, prior research also demonstrates that the content of users’ posts often becomes aligned with the content of the network they are embedded within (24). In tandem, these observations suggest that users embedded in more ideologically homogeneous and more hostile networks are more likely to increase their activity as well as their hostility in the face of divisive offline events. We already know that out-group animosity affects engagement on social media (22), but if echo chambers are also those with the greatest capacity for mobilization, then this would seem to further the potential negative downstream consequences of such networks. Specifically, these considerations entail the following prediction:

- H2.3: The effect described by H2.1 and H2.2 should be larger in more politically hostile and more ideologically extreme networks.

In tandem, hypotheses H2.1, H2.2, and H2.3—and the associated discussion—entail that the behavior of social media users at a given time reflect a dynamic interaction among offline events, the composition of the users’ online networks, and the users’ own dispositions. Offline events provide an impetus, and the offline network provides the exact social incentives that determine how individual users react, depending on their dispositions. In a final explorative test, we will formally examine the potential existence of a three-way interaction among all the key components of existing theoretical approaches: events, networks, and users.

Our third, and final, hypothesis focuses on the long-term consequences of the discussed dynamics. Research on echo chambers often highlights how ideologically homogeneous mindsets are a key driver of hostility because of the way that these mindsets shape information exposure and interpretation (7). However, other research shows that negative emotions in the wake of major political events, such as terrorist attacks or the COVID-19 pandemic, often strengthen in-group identification (46, 47). Thus, a potential consequence is that the online hostility generated by offline political events may also shape the users’ political identities in the longer run and leave people more mobilized for politics than before. As such, the online hostility triggered by offline events may over time increase ideological homogeneity of the individual social media user. Specifically, we hypothesize:

- H3: The political hostility of users may increase the ideological homogeneity of their social media posts.

If this conjecture is valid, it has important implications. If politically hostile content is a major driving force behind ideological homogeneity on social media, this could suggest the existence of self-reinforcing feedback loops where hostility-generating offline events strengthen online echo chambers and, hence, increase the hostility generated by the next event. As individuals become more ideologically homogeneous, the processes of selective exposure and interpretation that pull people toward online echo chambers are likely to intensify (25, 48). In that sense, divisive political events may play an important role in the strengthening of echo chambers and in their impact on online political interactions.

**Fig. 1.**
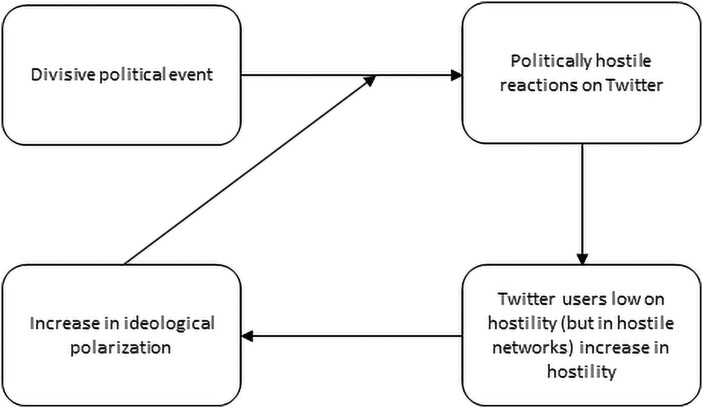
The potential hostility–homogeneity feedback loop initiated by offline divisive political events.

This potential process is illustrated in Figure 1. The figure displays a feedback loop that, everything else equal, becomes reinforced over time: If users become hostile as a consequence of divisive political events, this leads to an increase in the users’ level of ideological extremity, which in turn makes users more hostile over time and so on.

## Data and methods

The context for our empirical tests of our three sets of hypotheses is the period leading up to, and immediately following, the 2020 US presidential election. This period is an important case for our investigation of the importance of divisive political events for the development of political hostility. In everyday life, many Americans are not overly interested in politics (49), but during election times or crises, more people become politically interested and attentive (50, 51). Likewise, news consumption on social media increases during election time (52). Therefore, we expect that this is a period where more people become attentive to politics and are more likely to be affected by political events. Accordingly, while some previous findings have found little support for chamber-like patterns in news sharing (15), this may be because they were not focusing on periods where divisive events come into play and, hence, when people are not oriented toward policies. This also implies that if we do find that there is a strong effect of divisive political events on the formation of an individual's political hostility, perhaps exacerbated by polarized and politically hostile networks, this is also where it would hurt the most: when citizens are actually paying attention to politics.

To ensure significant diversity in the participants in this study, we rely on a unique data infrastructure whereby participants were first recruited by a professional survey agency and signed up for the collection of their online behavioral Twitter data via participation in the survey; descriptive statistics and demographics can be found in Table S1 in Appendix A. As described below, this setup also provides additional advantages through the combination of survey and behavioral data. Specifically, we commissioned the survey firm YouGov to invite panelists from their existing online panel. In January 2019, we gathered the first round of consent and added an additional group of participants in September 2020 to boost the study's power. The consent ended in January 2021. The number of respondents for the analyses that only use Twitter data is 2,834 for the studied time period. For the analyses where we also use survey data, there are 2,567 respondents since we were not able to link all survey responses to their Twitter handle. For these panelists, we also collected data on who their friends and followers were (*N* = 2,707,962) and also collected tweets from these Twitter users (roughly 2 billion tweets).

The project complies with the European General Data Protection Regulations according to the Tech Transfer Office at Aarhus University. Furthermore, as specified in the Danish law of the National Committee on Health Research Ethics (§14.2), surveys “that do not involve human biological material” are exempt from further ethical review.^1^

### Methods

All analyses are conducted in Mplus version 8 (53) using the MplusAutomation package (54) in the statistical software R (55). All graphs use the ggplot library (56) in R.

We use robust standard errors to account for potential nonnormality of our distributions when estimating standard errors. We use full information maximum likelihood to estimate missing values (57).

All models estimated are using the longitudinal aspect of the data. When estimating the effect of events and the moderating effects of the network, we are using each respondent as her own control.

This allows us to hold many time-constant constructs constant, such as stable predispositions, which might confound our estimates of the importance of the network and the importance of events. In addition, the attack on Congress can be viewed as a highly exogenous event, since it, for the majority of Twitter users, was a complete surprise (this is also evident in the figures below, demonstrating a clear spike in the development of political hostility at the very day of the attack). More formally, our model can be written:


$$Y_{it}=\alpha_{i}+\beta \cdot EventDummy_{it}+\epsilon_{it}$$

*Y_it_* represents the dependent variable of interest for panel unit *i* at time *t*; *α* represents unobserved differences between individuals; *EventDummy* is the dummy variable for the Event, i.e. the murder of George Floyd or the attack on the Congress for panel unit *i*; *β* is the estimated coefficients for the event dummy; and *ɛ* represents the error term capturing unobserved factors or random variation.^2^

By including fixed effects for each individual, the fixed effects model accounts for unobserved individual-level factors that may influence both the treatment (or time) and the outcome variable. As a result, the model can better isolate the causal effect of the treatment variable on the outcome variable by removing the confounding effects of time-invariant individual characteristics.

Controlling for individual fixed effects reduces the variation in the outcome variable that is attributable to individual-specific factors, making the remaining variation more likely to be due to the treatment variable or other time-varying factors. This leads to more efficient estimation and smaller SEs compared with a pre–post model that does not account for individual-level nesting.

There are nonetheless limits to how strongly the effects are causally identified. The most important challenge here is endogeneity. Two important limitations would be pretreatment trends and day-of-the-week effects (59). To investigate day-of-the-week effects, we include Figures S10 and S11 in Appendix A and find no clear evidence of day-of-week effects. To investigate pretreatment trends, we include Figures S8 and S9 and find some evidence for a pretreatment trend for the murder of George Floyd but not for the attack on Congress.

Although we thus do not find any clear evidence of pretreatment trends or day-of-the-week effects for the attack on Congress, and although we are able to find almost identical effects across two events, endogeneity obviously cannot be ruled out in a nonexperimental setup. Further studies using randomized controlled experiments in a fully controlled setting are in the end necessary to draw firm causal inferences.

### Measures

There is little agreement on either the definition or measurement of online hostility, which is a challenge for current approaches to capture this phenomenon (3). We therefore use a novel word-embedding approach to define and measure online political hostility (8). The basic idea behind the methodology is that it allows us to measure what participants *perceive* as political enmity based on the language they use surrounding the words “political” and “hate.” Four phases make up the approach as it is employed here. First, we build word vectors based on tweets from our panelists and the tweets in the network using the word2vec approach (60). The numerical representation of a word in a multidimensional space is called a word vector. As a result, word vectors can be used to calculate the distance between words. For instance, how closely related are the words “political” and “hate”? Second, we create a combined “political hate” word vector by joining the words “hate” and “political.” Third, we determine the separation between each tweet and the political hate word vector using the cosine similarity measure. A tweet is more politically hostile if it contains a lot of words that are near to the “political hate” word vector; less politically hostile tweets contain less words that are close to the word vector. The political hate scores of a respondent's tweets are averaged to generate a measure of their overall level of political hostility. We have termed this approach a super-unsupervised approach to acknowledge that it is not quite supervised and no external annotators are used but not quite unsupervised either, since the researcher herself chooses the particular “label” to be used in the study.

It might seem counterintuitive that we can simply use the two words “political” and “hate” to measure political hostility, but the approach has been subjected to a long series of robustness tests. First of all, expert raters were used to validate the procedure. Here, expert raters were given the following definition of online hostility: “We define *online political hostility* as the expression of degrading, malicious, and intimidating online behavior toward individuals or collectives based on political differences.” Using scores from the super-unsupervised word embedding approach and comparing them with the expert-rated tweets, we obtained an accuracy of 74 against the raters of experts (8). Furthermore, political hostility correlates with self-reported hostility (0.24), toxicity scores (0.47), sentiment scores (−0.32), political knowledge (0.35) and political interest (0.33). And, the “political” vector correlates higher with the political elements, compared with the “hostility vector,” whereas the “hostility” vector correlates higher with the hostility measures, compared with the “political” vector. These findings testify to the method's, convergent and discriminant validity. The method is also subjected to a series of tests of criterion validity, external validity, and ecological validity—see Rasmussen et al. (8).

A distinct advantage of the present measure of online political hostility, together with other recent measures (10, 61), is its ability to directly capture the *political* dimension of online hostility. Prominent measures of online hostility such as toxicity scores (62) or sentiment scores (63) are not able to do this, and part of the reason why previous studies have had difficulties in clearly linking online political hostility to distinct events could be measurement related (37). We measure hostility among panelists and in their network as identified by their friends and followers.

*Ideological diversity* (and its counterpart, ideological homogeneity) is also measured using behavioral Twitter data (i.e. text) in our panel and in the network. The most popular current measure toward measuring *network* ideology is based on Pablo Baberás novel approach (64), which estimates an *individual's* ideology based on whether they follow or befriend known political actors with clear ideologies. This approach has also been used to measure the ideology of an individual's network (24), although the method was not developed or validated for this. For our purposes, this approach is not fine-grained enough since we are interested in *changes* in ideological diversity on a monthly or potentially daily basis, whereas Barbera's network approach toward measuring ideology only changes if a person drastically changes the *composition* of the network and does not reflect changes *over time* within the same network. First, we used the survey data from the panelists to divide all panelists into either Republicans or Democrats using the standard seven-point question measure of Party Identification from ANES ranging from “Strong democrat” to “Strong Republican”; the middle category “Independent” is removed. We then created a balanced data set of tweets from Republican and Democratic panelists and used the popular BERT model to create a classifier to predict whether a tweet was Republican or Democratic (65).^3^ We obtained a test accuracy of 82% (see the full confusion matrix in Table S11 in Appendix A). We used this classifier to classify all tweets among panelists and in the network as either Republican or Democratic. Ideological diversity refers to the standard deviation of this measure in the network: If the standard deviation is high, there are tweets from both Republicans and Democrats; conversely, if the standard deviation is low, there are mainly tweets from one ideological side. To facilitate interpretation in some later analyses, we utilized a reverse-coded version of this measure to capture ideological homogeneity. This measure is simply the opposite of the diversity measure: the more ideologically diverse, the less homogeneous.

## Results

### Testing H1: over time changes in political hostility in social media networks during the 2020 US election

**Fig. 2.**
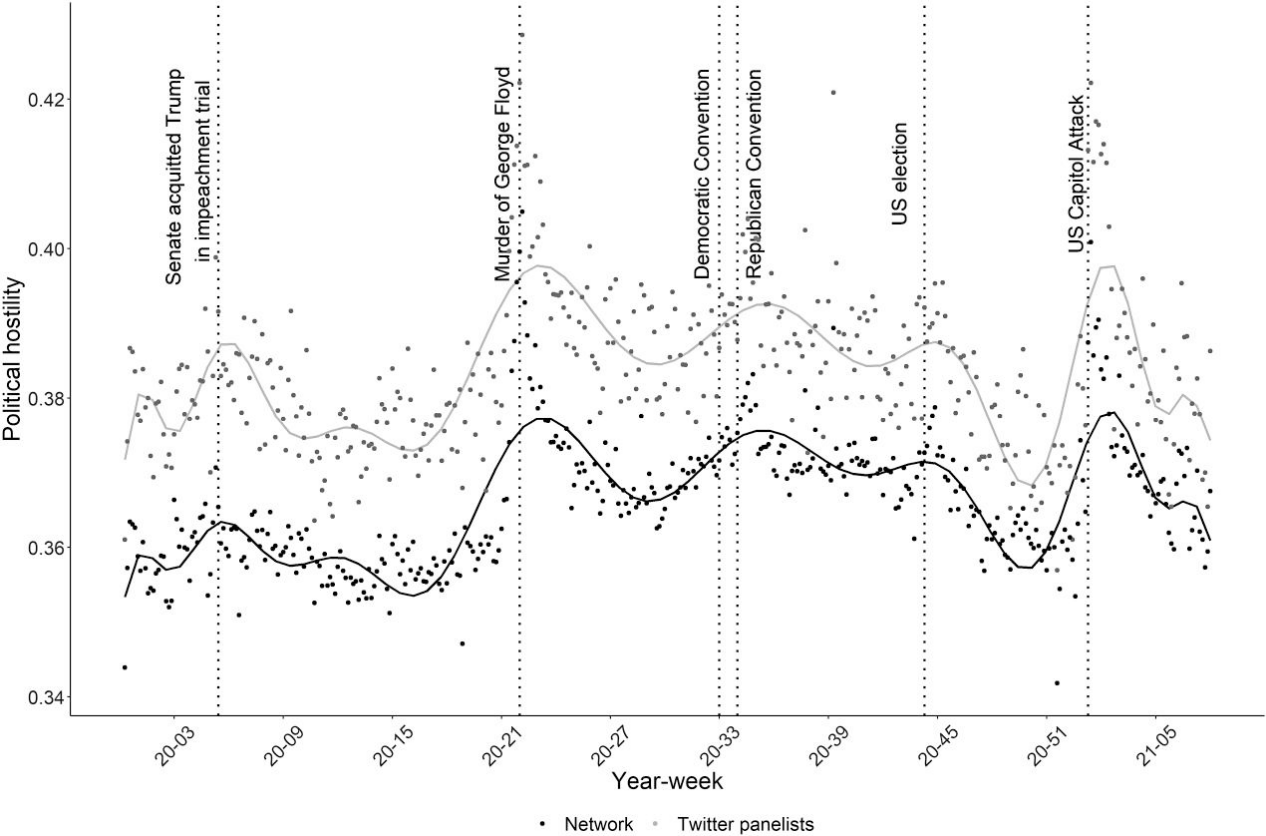
Development of political hostility in the United States for the panelists and the network of the panelists. The figure uses data on political hostility from our Twitter panelists and the political hostility from the network of our Twitter panelists. The Twitter data are first grouped by day and then an average is calculated. In order to display the data, we then used the R package ggplot2 (56) and plotted each individual point for this time period and an empirical smoothing corresponding to a 20th degree polynomial.

These results concern H1, i.e. whether temporal changes in political hostility in social media networks are clearly linked to identifiable events or not. Figure 2 illustrates the development of political hostility for both of our panelists and the network. The development of political hostility in the network and panelists follows an almost identical pattern in terms of temporal dynamics. In addition, as suggested above, the developments are highly event-driven. In fact, *all* major increases over the entire 2020 election in political hostility can retroactively be attributed to distinct political events. Thus, although previous studies have focused on social media networks as closed systems with their own dynamics and developments, when it comes to the development of political hostility, *all* major changes seem largely driven by events outside the system itself. This provides empirical support for H1.

The two largest peaks concern the murder of George Floyd (2020 May 25) and the attack on Congress (2021 January 6); see also Kim (27) and Jakubik et al. (31). In the following, we focus on these two events as drivers of political hostility since this is where the largest peaks are; if events do not matter for the development of political hostility here, they are not likely to matter for less influential events. Furthermore, the attack on Congress is a particularly clear exogenous event, and, hence, the causal effect estimate of political events on the development of (an individual's) political hostility is more robust.

### Testing H2.1 and H2.2: the direct effect of divisive political events on political hostility and activation

**Fig. 3.**
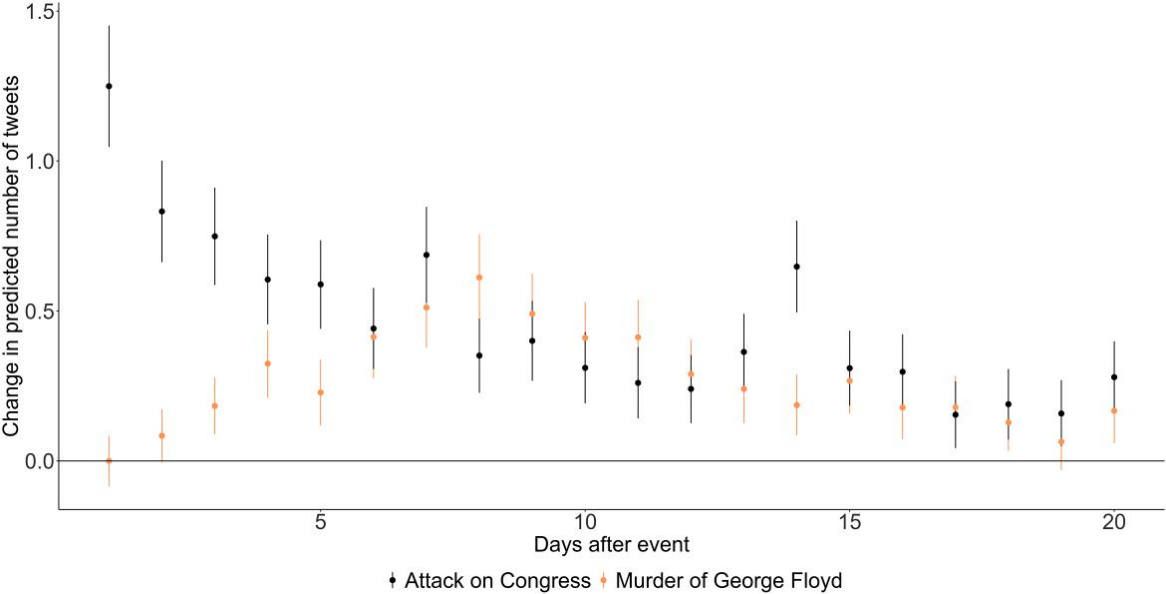
The effect of the attack on Congress and the murder of George Floyd on number of tweets. The figure illustrates the effect of the attack on Congress and the murder of George Floyd using day of year dummies, i.e. a dummy for the day before the attack and then running separate models for the subsequent days, including only the day before and the comparison day.

To investigate H2.1, i.e. whether divisive events activate social media networks, we examine whether the number of tweets increases following political events in Figure 3. For the attack on Congress, we see an immediate increase in the (intraindividual) number of tweets for the panelists, whereas there is a gradual build-up for the murder of George Floyd. This suggests that a first step in the wake of divisive political events is the activation of networks of friends and followers. This provides support for H2.1.

**Fig. 4.**
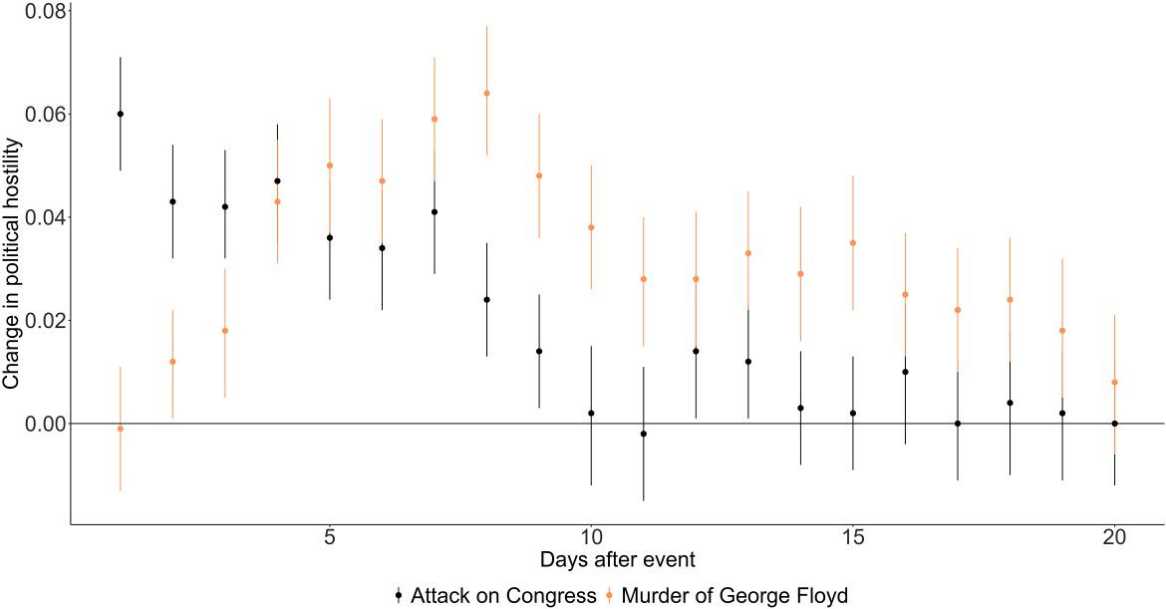
The effect of the attack on Congress and the murder of George Floyd on political hostility. The figure illustrates the effect of the attack on Congress and the murder of George Floyd using day of year dummies, i.e. a dummy for the day before the attack and then running separate models for the subsequent days, including only the day before and the comparison day.

Figure 4 illustrates a similar pattern as for tweeting but focuses on increases in political hostility.

Again, we observe an immediate effect for the attack on Congress and a more gradual build-up for the murder of George Floyd. The effect is roughly 0.06 on a scale from zero to one for the attack on Congress on the first day, and the highest effect size for the murder of George Floyd is roughly the same size. The combined effect of these small increases is quite visible in the initial Figure 2 for both the network and the group of panelists.

These observations are thus consistent with H2.2.

**Fig. 5.**
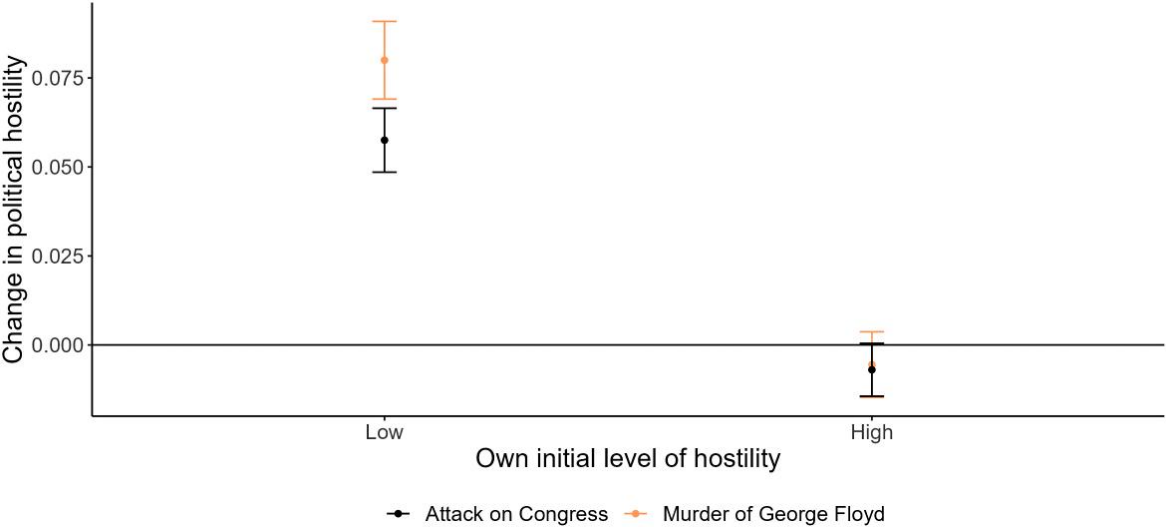
The effect of the attack on political hostility as a function of own initial level of hostility. To estimate this model, we are using the same specification as for the simple before-after model but use a window of 5 days before and 5 days after to be able to include as many panelists as possible, while also staying as close as possible to the event, since not every panelist tweets every day. In Figures S10 and S11, we demonstrate that this time period is a sensible time period since it is close to an average effect when varying the time points before and after the event for both events. The estimated parameters needed to reproduce the figure can be found in Table S2 in Appendix A. We investigate the robustness of choosing only two categories in Table S3 in Appendix A and find that it is robust to choosing three categories.

As an additional analysis, we investigate whether this effect is present for all or depends on user's own initial level of hostility. Specifically, we created a dummy variable to indicate whether the level of hate the day before the event was above or below the mean hostility for all panelists. Figure 5 illustrates that divisive political events push those who are low on political hostility higher on political hostility, whereas it does not affect those who are already high on political hostility.^4^

### Testing H2.3: the moderating effect of networks in the face of divisive events

The previous results are suggestive in terms of how divisive political events activate users, which in turn makes these users more politically hostile. In the following, we therefore investigate whether political hostility and extremity in the *network* amplify the effect of events on (individual-level) hostility and mobilization efforts.

#### Methods

We use the average of the preceding month of network data to quantify which type of network the panelist typically resides in, i.e. whether it is, on average, hostile/polarized or not. All models investigating the hate in the network are thus multilevel models, as above, but here the network is treated as a level 2 variable.

When estimating interaction effects, we are simply extending the model to include the moderator, the “main effect,” and the interaction between the event dummy and the moderator, the “interaction term.” When estimating the three-way interactions, we follow the usual procedure by including all lower order interactions. Full parameter estimates for these parameters can be found in Appendix A, where the tables underlying the figures can be found. All models investigating political hostility as a dependent variable are standard SEM models assuming multivariate normality. The models estimating the number of tweets are estimated in an identical manner but do not use a linear model assuming multivariate normality. Instead, we are using a model appropriate for modeling counts, while also allowing for the variance and mean to differ, and we therefore model these using a negative binomial regression (66).

#### Results

**Fig. 6.**
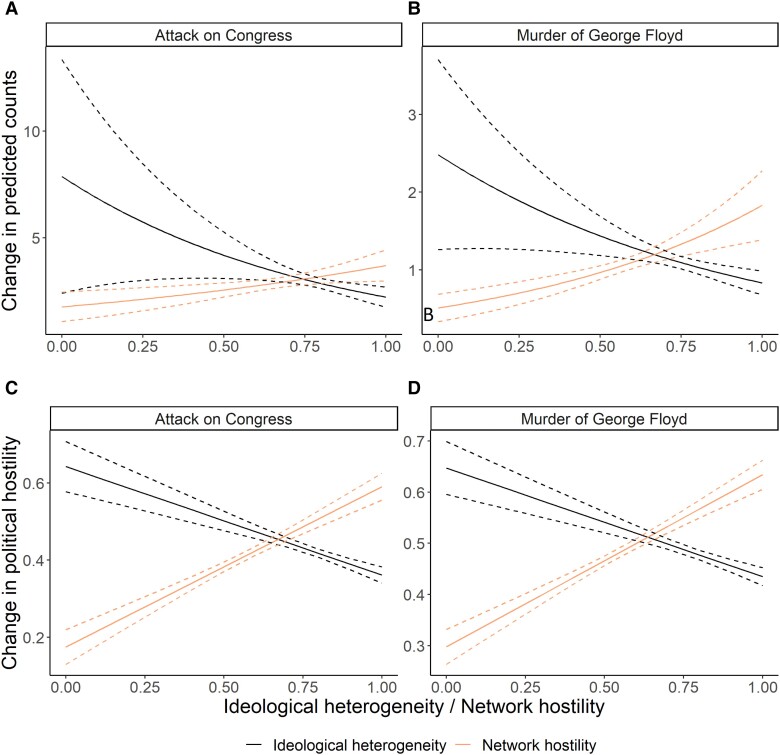
The effect of the attack on Congress and the murder of George Floyd on the number of tweets (the top two graphs) and political hostility (the lower two graphs) as a function of ideological diversity and political hostility in the network. The model estimated for political hostility is the same as for Figure 5 and also uses the same window size, i.e. 5 days before and after, except it also includes an interaction term, and main effect, for the network's ideological diversity or hostility. Next, we calculate predicted values for those low on political hostility, i.e. a value of zero, by simply plugging values into the parameter estimates obtained in Table S6 in Appendix A. Exactly the same approach is used for the predicted counts except here we have a binomial regression.

In the top two graphs in Figure 6, we can clearly see that in the most hostile and polarized networks, the number of tweets following these two divisive political events are much higher compared with those residing in ideologically diverse networks or in networks with little political hostility. For instance, following a divisive political event, the predicted number of tweets for a person in a highly hateful network is roughly two to three tweets, whereas it is less than one for a person in a nonhostile network. This is a huge effect; an effect more than quadrupling the number of tweets potentially means thousands, or millions, of more tweets writ large over the entire Twitter network. Summing up, following divisive political events, there seems to be a large mobilizing effort in the most hateful and polarized parts of the Twitter networks.

The second analysis concerns whether these two divisive events also led to more hostile tweets among users embedded in ideologically diverse or hostile networks. If the increased activity is not related to increased political hostility, the activation may be less normatively problematic, and conversely, if the activation does indeed come with more hostility, this suggests that hostile and homogeneous networks may either negatively shape the behavior of social media users or be particularly attractive to social media users in times of turmoil.

The two lower graphs in Figure 6 show the predicted values for those initially low on political hostility as a function of (i) ideological diversity and (ii) hostility in the network. The moderating network effects, i.e. ideological diversity and political hostility, are of roughly the same size. The effects of the network on the development of political hostility are quite large: The predicted level of hate following the murder of George Floyd for those who are lowest on network hostility is roughly 0.2, whereas it is roughly 0.6 for those in the most hateful network. Thus, the effect is almost three times as big for those in the most hateful network. Or put differently, going from those in the least hostile networks to the most hostile networks increases the predicted level of political hostility by 0.4. These are obviously maximal effects and larger than the average effects obtained above in Figure 5 of around 0.075. The mean network hostility level is 0.574 with a standard deviation of 0.134. Since most respondents likely fall within the range of ±2 standard deviations, most respondents range between 0.306 and 0.842. This roughly corresponds to an effect increase between 0.4 and 0.6, i.e. an increase of roughly 0.2 is thus only slightly more than double the average effect from Figure 5 above.^5^

When social media users on Twitter experience divisive political events, such as the murder of George Floyd or the attack on the US Congress, individual-level hostility increases—this applies mostly to those who are in the most polarized or hostile networks, presumably due to these networks’ larger activation efforts. We can thus confirm H2.3.^6^

As a final analysis, we have combined the observations above and estimated models including three-way interactions between the network and one's own initial level of hostility. These three-way interactions are significant for both the attack on Congress and the murder of George Floyd. For those who are initially high on political hostility, there is no effect when divisive political events occur, no matter how hostile or polarized your network is. However, for those who are initially low on political hostility, the network matters a great deal, and they become as hostile as those who are initially high on political hostility. See Figure S12 in Appendix A.

### Testing H3: political hostility and political homogeneity: political hostility drives political homogeneity

The above sets of analyses have shown that hostile networks amplify political hostility in the face of divisive events and that such events and networks push those who are initially low on political hostility to be as high on political hostility as those who are initially highest on political hostility. Furthermore, we found that these effects were particularly pronounced in politically hostile and homogeneous networks. In the last set of analyses, we asked whether this increased hostility has downstream consequences by subsequently increasing ideological homogeneity and, potentially, facilitating the further formation of echo chambers.

#### Methods

The close associations between political hostility and ideological homogeneity raise questions about directions of influence. Are ideologically homogeneous networks on social media influencing political hostility or the other way round? To examine this, we have estimated a random intercept cross-lagged panel model (RI-CLPM), focusing on political hostility and political diversity (i.e. the reverse-coded measure of ideological diversity). The model incorporates both stability and change indicators. Positive stability scores suggest that those who were (politically hostile or diverse) at *time1* were also more hostile or diverse at *time1 + 1* relative to their own expected mean. In contrast, reciprocal effects, such as the effects of political hostility on political diversity (online hostility → diversity) or diversity on hostility (polarization → online hostility), show that a (positive) deviation from an individual's expected mean in political diversity or hostility also leads to a (positive) deviation in the expected mean of political diversity on online hostility. The marginal Spearman correlation between these two measures is −0.27, which initially suggests a negative correspondence between how diverse a network is and how hostile it is; the more hostile, the less diverse. The RI-CLPM thus enables us to dig deeper into the reciprocal relationships between ideological diversity and online animosity. That is, we can ask whether increased ideological diversity leads to increased online hostility or whether online hostility leads to increased ideological diversity.

Classical cross-lagged models have been chastised for failing to discriminate between variation *between* individuals and variation *within* individuals because the parameter estimates from such a model are a mix of the two (67). In contrast, the RI-CLPM divides the variance into separate estimates for between and within variance. This method, which is similar to a fixed effect model in which the (within-group) mean is subtracted to isolate within-individual variation, is achieved by estimating a random intercept for each construct such that the cross-lagged parameter estimates are based solely on within-individual variation. This allows us to hold constant time-constant variation such as general dispositional traits, e.g. in the form of personality traits. As mentioned in the Introduction, one of the major strands of research attempting to explain differences in online political hostility to differences in personality traits. A model wishing to explain the reciprocal relationship between online political hostility and homogeneity therefore needs to take this aspect into account. Conversely we cannot, using the RI-CLPM model, directly test or investigate the second strand of literature stressing the role of networks. Since the RI-CLPM model that we use here only investigates individual-level changes in two constructs, we are not directly modeling how network composition or selection might affect the results. Further studies using other models and measures are needed to take this perspective into account.

Compared to the results in Figure 4, which are based on a more classical pre–post comparison, these results draw on an observational statistical model with important limitations and caveats. First, our main interest is the *direction* of influence, i.e. from political hostility to homogeneity or from homogeneity to political hostility. Second, we are not claiming that the model is able to draw clear causal inferences using observational data, but that we attempt to model the relationship between two important constructs over time taking into account important confounders in process, notably stable individual predispositions such as personality traits. The RI-CLPM model is ideally suited for studying changes over time, while taking time-constant variation into account, but obviously time-varying confounding cannot be ruled out (68) and would lead to a misspecified model. In addition, the model cannot, and does not claim to, model contemporaneous effects (69). If a researcher is therefore interested in modeling contemporaneous effects, another model should be used.

In the following, we use all the individual Twitter users in the network. The reason for using all individual users in the network is a simple power consideration: In the time period studied below, August 2020 to December 2020, where we have full data on all tweets from the network, we have 603,944 unique users as opposed to the 2,834 panelists used above. We do not have full network data on the period including the attack on Congress and therefore cannot include this in the model. Since we are not investigating the effect of specific events, but using a RI-CLPM model that investigates the average (individual-level) changes over time, this should matter less as we are not suggesting that the longitudinal dynamics should somehow only matter at specific time points.

In this period, we have collected 453,326,975 tweets from these individuals. The number of observations in the RI-CLPM model for this period is 530,955. To get a precise and detailed measure of the ideological homogeneity and political hostility, we have grouped the tweets by months, and we are thus investigating 5 months of data. In addition, we have removed outliers since the RI-CLPM relies on normally distributed variables (70). The results without removing outliers can be found in Table S10 in Appendix A.

#### Results

**Fig. 7.**
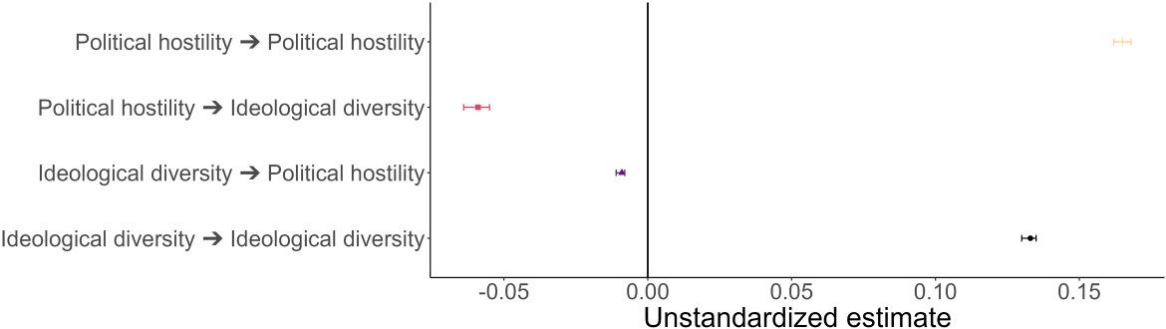
RI-CLPM for political hostility and political diversity.

Figure 7 illustrates that both constructs influence each other, but the effect of political hostility on political polarization is larger than the reverse effect. This suggests that Twitter users who were hostile in the past are more likely to be politically polarized in the future. A deviation from the person-specific mean in political hostility is associated with a deviation (from the person-specific mean) in ideological diversity of −0.059 (95% CI −0.064; −0.055), and the reverse effect is less than half of that −0.017 (95% CI −0.019; −0.014). The relationship is negative since ideological diversity measures how ideologically diverse the tweets are, and it seems that those who are more politically hostile are less diverse in their tweets. Therefore, we find support for H3.

## Discussion and conclusion

What drives hostility in political discussions on social media? While prior studies have highlighted the characteristics of users or their networks as key in explaining the dynamics of hostility, we have added to an emerging literature about the importance of temporal dynamics in online political hostility and how these dynamics closely follow the emergence of divisive offline political events. We investigated this using Twitter and the US 2020 election year as cases and demonstrated that central divisive events during this year (specifically, the murder of George Floyd and the attack of US Congress) led to increased Twitter activity and increased political hostility. Hostility following divisive events was more strongly expressed by individuals in politically hostile and homogeneous networks, but these events also reconfigured the composition of hostile individuals, such that individuals not already engaged in hostility became mobilized. These findings suggest that users, networks, and offline events cannot be analyzed in isolation: Political hostility emerges as a dynamic interaction between all components.

Finally, we found that this increased hostility has long-term consequences by increasing the homogeneity of user's subsequent activity, potentially increasing the likelihood that they select into homogeneous networks or echo chambers. As such, divisive events may not only influence the composition of hostile users but may also facilitate the formation of echo chambers. This potentially entails a feedback loop where some users become more likely to respond with hostility to the next divisive event.

To establish these results, we have taken a number of steps to increase the internal validity of the analyses. At the same time, as noted above, there are a number of challenges to causal inference for both the fixed effects models and for the RI-CLPMs. As such, while the analyses are still superior to traditional observational studies, we are not able to provide strict causal evidence.

Another important limitation concerns the concept of a “divisive event.” We have relied on an exploratory approach to identify divisive offline events. For further studies, it would be helpful to develop a conceptualization of divisive events that allow for a priori assessment of whether an event is divisive or not. A more robust examination would use such a conceptualization to develop a corpus of divisive events and analyze their effects on hostility across cases. In this regard, it should also be noted that not all events may shape online political hostility in the same way as we have identified here. Prior research suggests that online hostile interactions may also increase the likelihood of offline activism including violence (30–32). As such, some divisive offline events may, in part, be the effects rather than the causes of online political hostility. Furthermore, while we found that both Democrats and Republicans responded equally to the events under study here, other events (e.g. less salient events) may exhibit greater partisan asymmetry in the attention they attract and the hostility they generate.

These limitations notwithstanding, the findings have important implications. The findings show that hostility has strong temporal dynamics. Methodologically, current empirical studies on echo chambers and ideological polarization implicitly assume that their effect-estimates, for the period of time their data cover, are representative of the typical inner workings of social media. Yet, even when the time-bound nature of effect-estimates is recognized (22), there is no way of knowing just how much the effect-estimates are affected. We demonstrate empirically that timing is crucial for the activation of political hostility. When effect-estimates are small, and when timing is not explicitly modeled (e.g. (71)), we urge caution in interpreting the results. This conceptual point might also help shed light on why some of the findings on hostility and echo chambers are mixed (14, 72). If they are studying a period where networks are not activated, we would indeed not expect social media networks to generate hostile reactions.

The temporal dynamics of hostility also has important implications for authorities and practitioners working to decrease the hostility of online discussions. The present findings provide strong evidence that timing is key for successful interventions against online hostility. On the one hand, interventions need not be constantly deployed but, on the other hand, it is important that they are implemented in the immediate aftermath of divisive events. This is especially the case because the long-term effects of such divisive offline events, if they activate online hostility, are to facilitate the conditions for further hostility through the formation of echo chambers.

Finally, we want to raise the possibility that the present findings could imply a change in the concept of echo chambers itself. Echo chambers have been defined by Jamieson and Cappella (73 p.76) as a “bounded, enclosed media space that has the potential to both magnify the messages delivered within it and insulate them from rebuttal.” Yet, the demonstrated responsiveness of online networks to offline events suggests that an idea of an “insulated” media space where network participants mostly seek out news and opinions that match their own priors may not capture the dynamics of the relevant networks accurately. A more apt description of social media networks, when overrun by external events, may be a “resonance chamber,” i.e. the acoustic design used for musical instruments, which intensifies sound waves entering the semiclosed system from the outside. We are not claiming that conceptualizing social media networks as echo chambers cannot be useful when describing day-to-day politics, but we suggest that when faced with strong external shocks, such as highly divisive political events, social media networks may be better described as resonance chambers, emphasizing their partial openness.

## Supplementary Material

pgad382_Supplementary_Data

## Data Availability

All data, scripts, and materials will be made available on OSF upon publication in a format where individual respondents will still remain anonymous. This means only aggregated data, such as e.g. political hostility scores but not individual tweets, will be made available.

## Footnotes

1
https://nationaltcenterforetik.dk/ansoegerguide/overblik/hvad-skal-jeg-anmelde.

2 Since we are estimating the model using Mplus, this effect is actually estimated in a structural equation modeling framework, but this way of presenting the model is easier to understand and is more familiar. The within effect is however identical to the effect presented here (58).

3 For a fuller discussion of this model and its underpinning, see Appendix A2.

4 Given that we rely on a data infrastructure that includes survey data, we are also able to repeat this analysis using self-reported measures of individual dispositions highlighted in the user-oriented literature on hostility. However, these measures are less ideal as we are investigating short-term fluctuations. The analyses in Figure 5 take advantage of the fact that we have fine-grained data on Twitter behavior on a day-to-day basis. For each of the two events studied in the paper, we can thus investigate whether the panelists were high or low on political hostility just before the event. In Appendix A in Tables S4 and S5, we repeat the analyses in Figure 5 with measures of antisocial personality traits often highlighted in the user-oriented literature. None of these analyses show a significant interaction with the events, suggesting that people high and low in antisocial dispositions do not react differently to them. Thus, we only observe the mobilization effect, when we use the measures of Twitter behavior just prior to the event. This could suggest that the individuals who mobilize in the face of divisive events are individuals predisposed for hostility (given their personality) but who for some reasons were temporarily demobilized prior to the event (as reflected in the fine-grained Twitter data).

5 Using the parameters from Table S5, we can, e.g. calculate the effect of 2 SDs below the mean, i.e. 0.306 as follows: predicted value = 0.342 − 0.045 + 0.195 · 0.306 + 0.142 · 0.306 = 0.400122.

6 The figures in Section A1 in Appendix A show there are no substantial differences between Democrats and Republicans when we subset to those self-identifying as Democrats or Republicans when it comes to the effect of activation or increases in hostility.

## Preprints

This manuscript was posted on a preprint: https://psyarxiv.com/vzu4q/.
