# Supplementary material for: The event-driven nature of online political hostility: How offline political events make online interactions more hostile
Source: PNAS Nexus. 2023 Nov 10;2(11):pgad382. doi: 10.1093/pnasnexus/pgad382 (PMC10681799; doi:10.1093/pnasnexus/pgad382)
Supplement: pgad382_Supplementary_Data [file pgad382_supplementary_data.pdf]

APSR Submission Template APSR Submission Template APSR Submission Template APSR Submission Template APSR Submission Template APSR Submission Template APSR Submission Template APSR Submission Template APSR Submission Template

APPENDIX A:ADDITIONAL RESULTS

TABLE S1. Descriptive statistics for the panelists

Construct	
Age$_{max}$	14
Age$_{mean}$	6.337
Age$_{min}$	1
Age$_{sd}$	2.898
Age$_{count}$	9871
Education$_{max}$	6
Education$_{mean}$	4.417
Education$_{min}$	1
Education$_{sd}$	1.339
Education$_{count}$	9871
Female$_{max}$	1
Female$_{mean}$	0.487
Female$_{min}$	0
Female$_{sd}$	0.5
Female$_{count}$	9871
Household income$_{max}$	16
Household income$_{mean}$	7.09
Household income$_{min}$	1
Household income$_{sd}$	3.517
Household income$_{count}$	9315
Party Identification$_{max}$	7
Party Identification$_{mean}$	4.837
Party Identification$_{min}$	1
Party Identification$_{sd}$	2.142
Party Identification$_{count}$	9871
Political hostility$_{max}$	0.725
Political hostility$_{mean}$	0.374
Political hostility$_{sd}$	0.081
Political hostility$_{count}$	5761

We have 6 waves of survey data and these descriptive statistics are an aggregate.

Age is measured in these groupings: 1 = 18-22, 2 = 23-27, 3 = 28-32, 4 = 33-37, 5 = 38-42, 6 = 43-47, 7 = 48-52, 8 = 53-57, 9 = 58-62, 10 = 63-67, 11 = 68-72, 12 = 73-77, 13 = 78-82, 14 = 83+

Gender is measured on a binary scale where 1 = Female and 0 = Male.

Education is measured using these categories 1 = No HS, 2 = High school graduate, 3 = Some college, 4 = 2-year, 5 = 4-year, 6 = Post-grad

Party Identification is measured using these categories 1 = Strong Republican 1 = Strong Republican, 2 = Not very strong Republican, 3 =Closer to the Republican party, 4 = Independent, 5 = Closer to the Democratic party, 6 = Not very strong Democrat, 7 = Strong Democrat

TABLE S2. Model output for figure 5

Parameter	Murder of George Floyd	Attack on Congress
Period dummy	0.083<br>[0.073;0.094]*	0.058<br>[0.049;0.066]*
Period dummy x Initial level of hate	-0.092<br>[-0.106;-0.077]*	-0.064<br>[-0.076;-0.053]*
Initial level of hate	0.199<br>[0.190;0.208]*	0.167<br>[0.159;0.175]*

TABLE S3. Robustness test of including an additional dummy variable for initial level of hate

Parameter	Murder of George Floyd	Attack on Congress
Period dummy	0.069<br>[0.056;0.081]*	0.091<br>[0.076;0.106]*
Period dummy x Initial level of hate,high	-0.089<br>[-0.105;-0.073]*	-0.114<br>[-0.133;-0.095]*
Period dummy x Initial level of hate,medium	-0.042<br>[-0.056;-0.028]*	-0.048<br>[-0.066;-0.031]*
Effect of Initial level of hate, high	0.230<br>[0.221;0.239]*	0.261<br>[0.251;0.272]*
Effect of Initial level of hate, medium	0.134<br>[0.127;0.142]*	0.146<br>[0.138;0.155]*

TABLE S4. Investigation of using agreeableness as a moderator

Parameter	Murder of George Floyd	Attack on Congress
Period dummy	0.036<br>[0.023;0.049]*	0.030<br>[0.018;0.042]*
Period dummy x Agreeableness	-0.004<br>[-0.022;0.014]	-0.015<br>[-0.032;0.002]
Initial level of Agreeableness	-0.005<br>[-0.023;0.013]	0.009<br>[-0.009;0.026]

We have used the Big Five Inventory (BFI) consisting of 44 items to measure the five major personality traits (John, Naumann, and Soto, 2008).The alpha reliability was 0.82 for agreeableness

TABLE S5. Investigation of using psychopathy as a moderator

Parameter	Murder of George Floyd	Attack on Congress
Period dummy	0.036<br>[0.024;0.048]*	0.028<br>[0.015;0.040]*
Period dummy x Psychopathy	-0.007<br>[-0.026;0.011]	0.001<br>[-0.017;0.020]
Initial level of Psychopathy	0.006<br>[-0.014;0.025]	-0.007<br>[-0.026;0.013]

The psychopathy measure from (D. N. Jones and Paulhus, 2014) using these items: I like to get revenge on authorities,I avoid dangerous situations,Payback needs to be quick and nasty,People often say I'm out of control,It's true that I can be mean to others,People who mess with me always regret it,I have never gotten into trouble with the law,I enjoy having sex with people I hardly know,I'll say anything to get what I want. Alpha reliabile was 0.63

APSR Submission Template APSR Submission Template APSR Submission Template APSR Submission Template APSR Submission Template APSR Submission Template APSR Submission Template APSR Submission Template APSR Submission Template

TABLE S6. Model output for lower two graphs in figure 6

	Network hate		Ideological diversity	
Parameter	Murder of George Floyd	Attack on Congress	Murder of George Floyd 1	Attack on Congress 1
Period dummy	-0.071<br>[-0.110;-0.032]*	-0.045<br>[-0.069;-0.020]*	0.216<br>[0.165;0.267]*	0.145<br>[0.111;0.179]*
Period dummy x moderator	0.270<br>[0.202;0.339]*	0.195<br>[0.151;0.239]*	-0.144<br>[-0.206;-0.081]*	-0.107<br>[-0.148;-0.065]*
Period dummy x initial level of hate	-0.123<br>[-0.141;-0.105]*	-0.088<br>[-0.101;-0.074]*	-0.111<br>[-0.124;-0.098]*	-0.077<br>[-0.090;-0.064]*
Moderator	0.145<br>[0.081;0.209]*	0.142<br>[0.095;0.188]*	-0.137<br>[-0.197;-0.077]*	-0.106<br>[-0.157;-0.055]*
Initial level of hate	0.184<br>[0.172;0.195]*	0.161<br>[0.152;0.170]*	0.209<br>[0.200;0.219]*	0.168<br>[0.160;0.176]*

TABLE S7. Model output for top two graphs in figure 6

	Network hate		Ideological diversity	
Parameter	Murder of George Floyd	Attack on Congress	Murder of George Floyd 1	Attack on Congress 1
Period dummy	0.564<br>[0.172;0.956]*	-0.674<br>[-1.020;-0.329]*	2.063<br>[1.367;2.759]*	0.909<br>[0.416;1.402]*
Period dummy x moderator	0.744<br>[0.179;1.308]*	1.279<br>[0.710;1.847]*	-1.268<br>[-2.156;-0.379]*	-1.094<br>[-1.751;-0.438]*
Moderator	3.496<br>[2.467;4.525]*	2.654<br>[1.571;3.738]*	-0.245<br>[-1.789;1.299]	-2.704<br>[-4.158;-1.251]*

TABLE S8. Model output for figure 12

Parameter	Murder of George Floyd	Attack on Congress
Period dummy	-0.125<br>[-0.190;-0.060]*	-0.069<br>[-0.101;-0.036]*
Period dummy x network hate	0.356<br>[0.251;0.461]*	0.239<br>[0.179;0.299]*
Period dummy x initial level of hate	-0.006<br>[-0.092;0.080]	-0.004<br>[-0.058;0.050]
Period dummy x initial level of hate x network hate	-0.183<br>[-0.311;-0.055]*	-0.139<br>[-0.227;-0.050]*
Initial level of hate	0.281<br>[0.188;0.373]*	0.293<br>[0.234;0.353]*
Network hate	0.186<br>[0.084;0.288]*	0.221<br>[0.163;0.280]*
Initial level of hate x network hate	-0.125<br>[-0.261;0.010]	-0.224<br>[-0.322;-0.126]*

APSR Submission Template APSR Submission Template APSR Submission Template APSR Submission Template APSR Submission Template APSR Submission Template APSR Submission Template APSR Submission Template

TABLE S9. Model output for figure 7

Parameter	Estimates
Ideological diversity ⟶ Ideological diversity	0.133 [0.130;0.135]*
Political hostility ⟶ Ideological diversity	-0.059 [-0.064;-0.055]*
Ideological diversity ⟶ Political hostility	-0.009 [-0.011;-0.008]*
Political hostility ⟶ Political hostility	0.165 [0.162;0.168]*

TABLE S10. Model output for RI-CLPM without removing outliers

Parameter	Estimates
Ideological diversity ⟶ Ideological diversity	0.082 [0.080;0.084]*
Political hostility ⟶ Ideological diversity	-0.072 [-0.084;-0.059]*
Ideological diversity ⟶ Political hostility	-0.001 [-0.001;-0.001]*
Political hostility ⟶ Political hostility	0.101 [0.099;0.104]*

TABLE S11. Confusion matrix for classifier to predict ideology based on tweets

		Predicted	
		Republican	Democrat
Actual	Republican	0.89	0.11
	Democrat	0.24	0.76

APSR Submission Template APSR Submission Template APSR Submission Template APSR Submission Template APSR Submission Template APSR Submission Template APSR Submission Template APSR Submission Template

FIGURE S8. Average number of tweets pr. panelist and average level of political hostility before and after attack on Congress

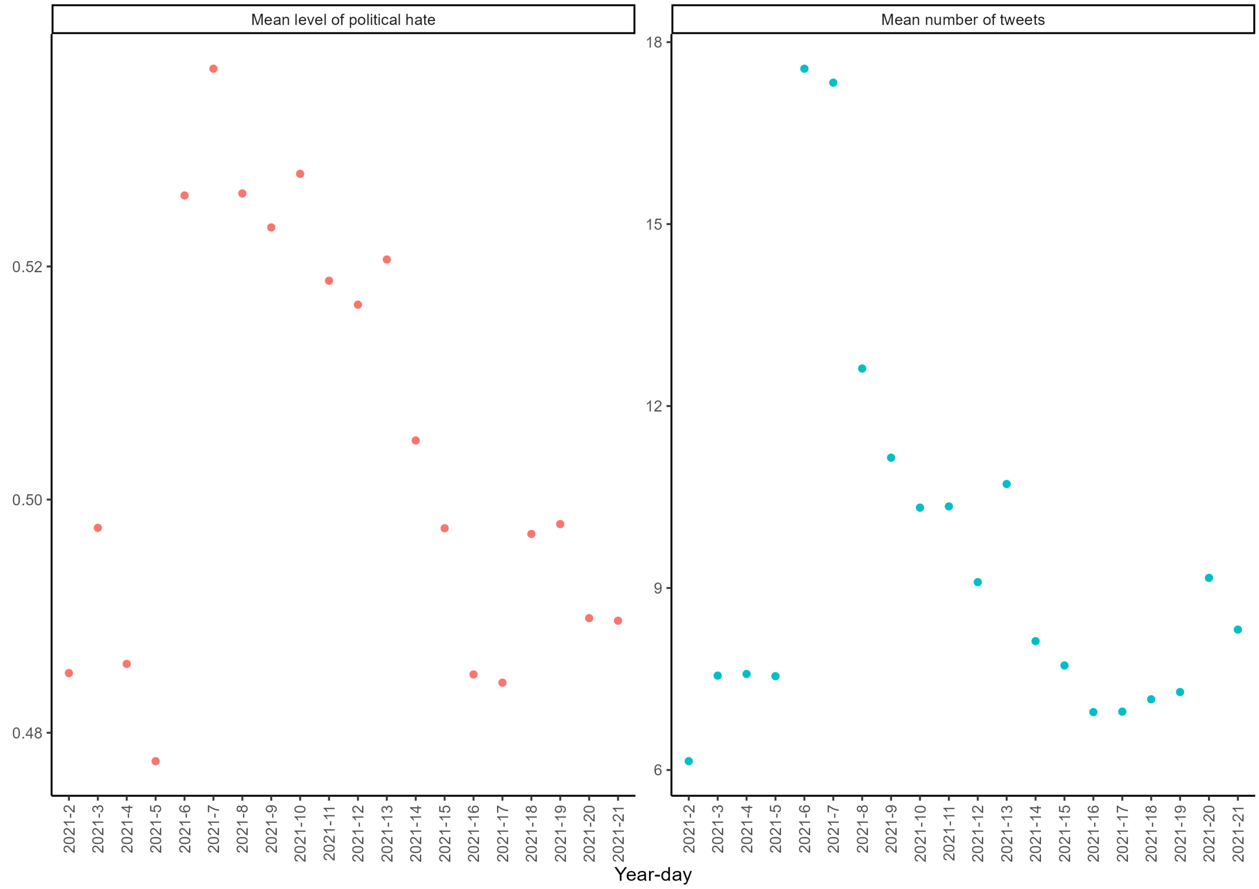

APSR Submission Template APSR Submission Template APSR Submission Template APSR Submission Template APSR Submission Template APSR Submission Template APSR Submission Template APSR Submission Template

FIGURE S9. Average number of tweets pr. panelist and average level of political hostility before the murder of George Floyd

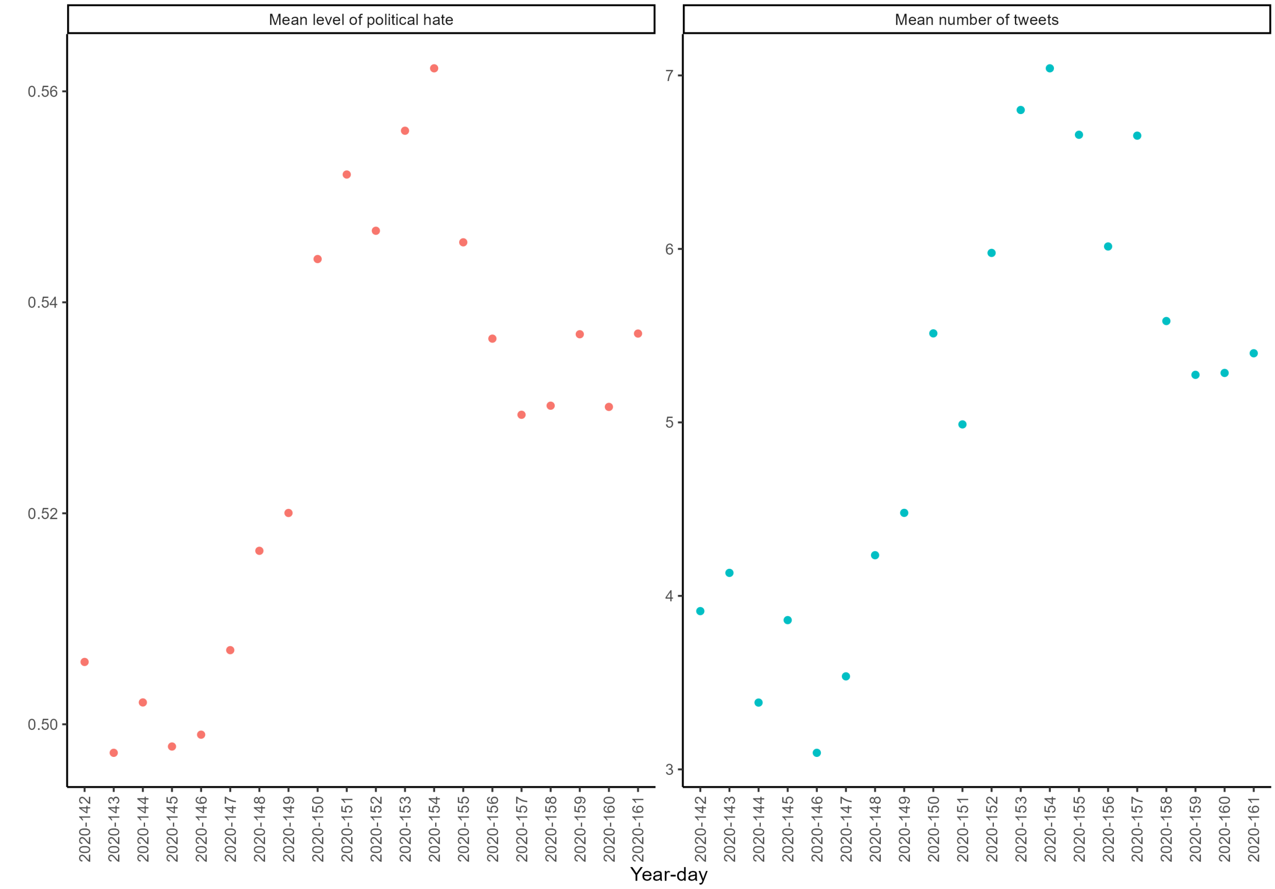

FIGURE S10. Effect estimate for main effect when varying the window before and after the event for the attack on Congress

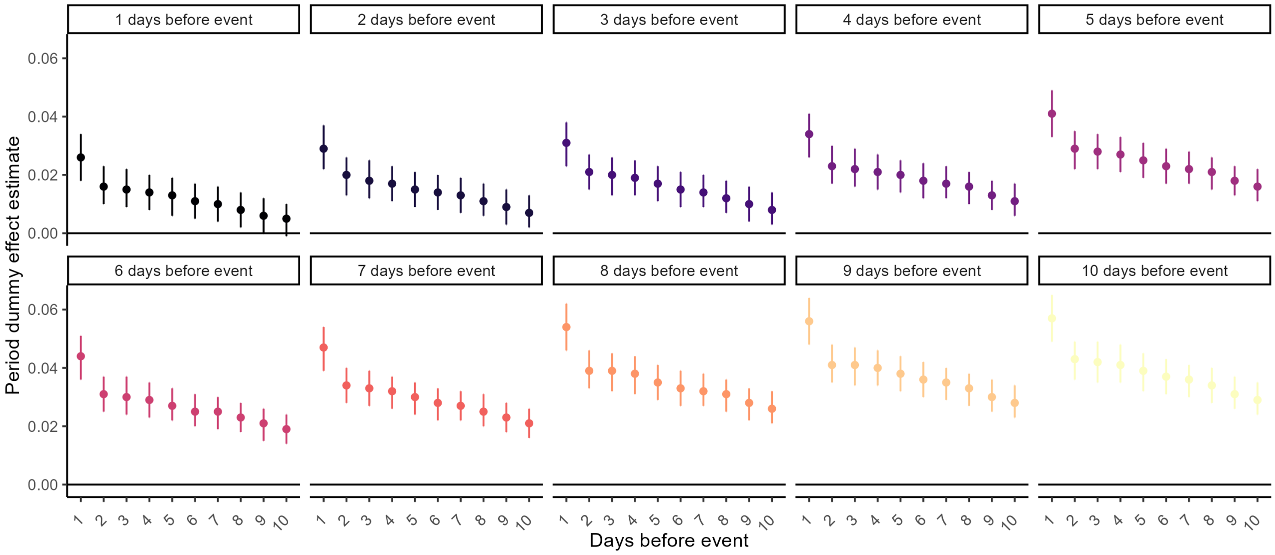

APSR Submission Template APSR Submission Template APSR Submission Template APSR Submission Template APSR Submission Template APSR Submission Template APSR Submission Template APSR Submission Template

FIGURE S11. Effect estimate for main effect when varying the window before and after the event for the murder of George Floyd

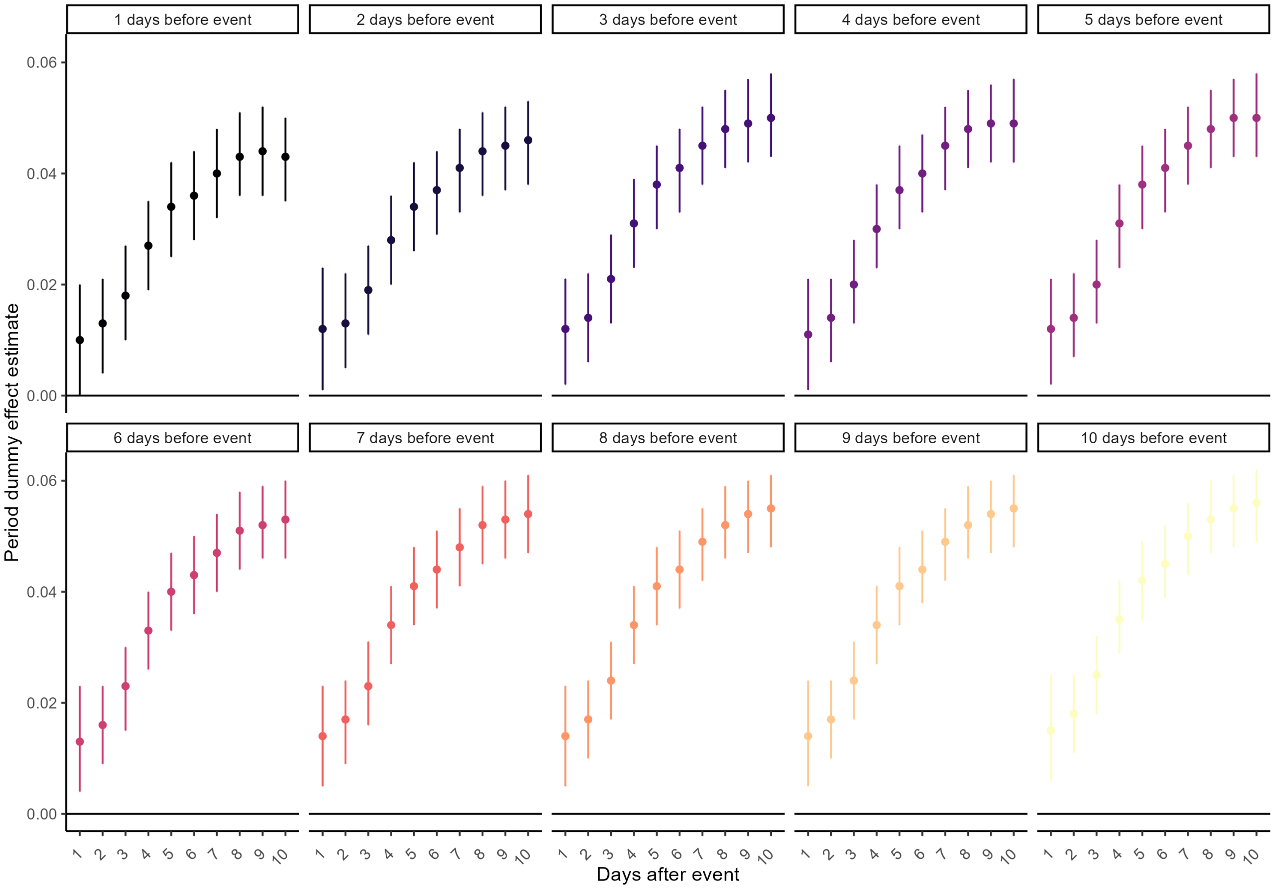

FIGURE S12. Effect of hostility as a function of political hostility in the network and initial levels of own political hostility

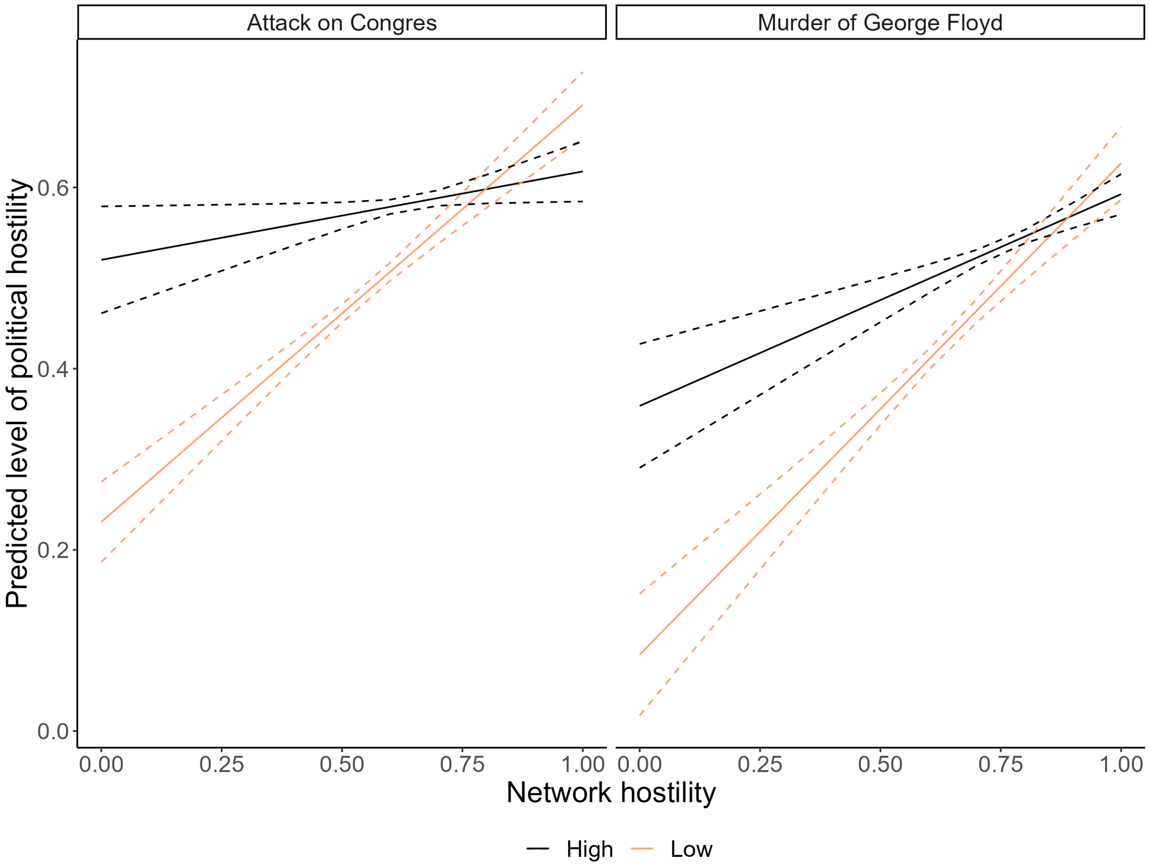

APSR Submission Template APSR Submission Template APSR Submission Template APSR Submission Template APSR Submission Template APSR Submission Template APSR Submission Template APSR Submission Template

A1: Results split by party identification

We use the standard seven-point question measure of Party Identification from ANES ranging from "Strong democrat" to "Strong Republican" where the middle category "Independent" is removed to group respondents into "Republicans" and "Democrats".

FIGURE S13. The effect of the attack on political hostility as a function of own initial level of hostility for those self-identifying as Republicans

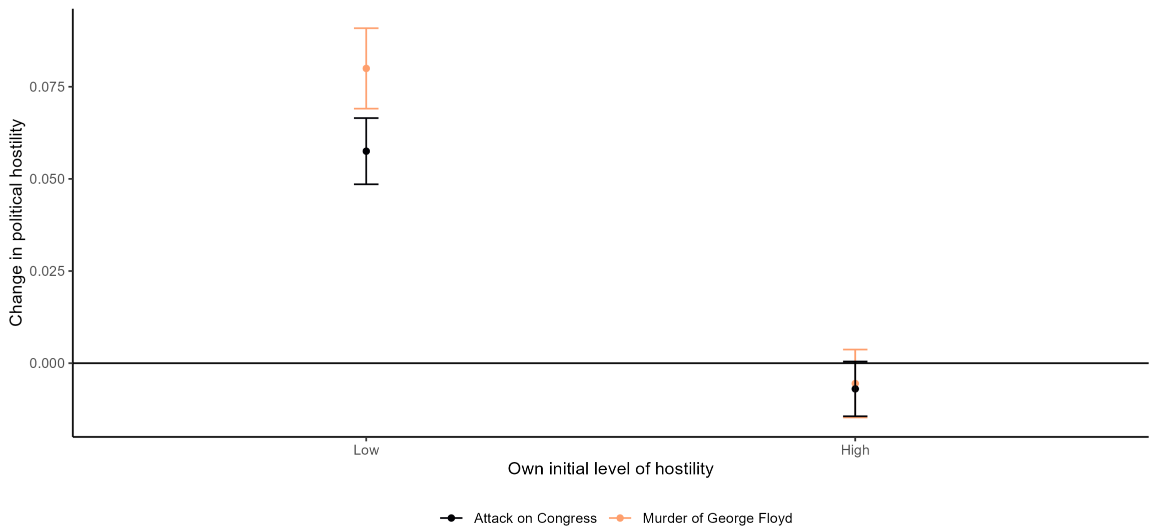

The figure illustrates the effect of the attack on Congress and the murder of George Floyd using a single time dummy for those initially low and high on political hostility

FIGURE S14. The effect of the attack on political hostility as a function of own initial level of hostility for those self-identifying as Democrats

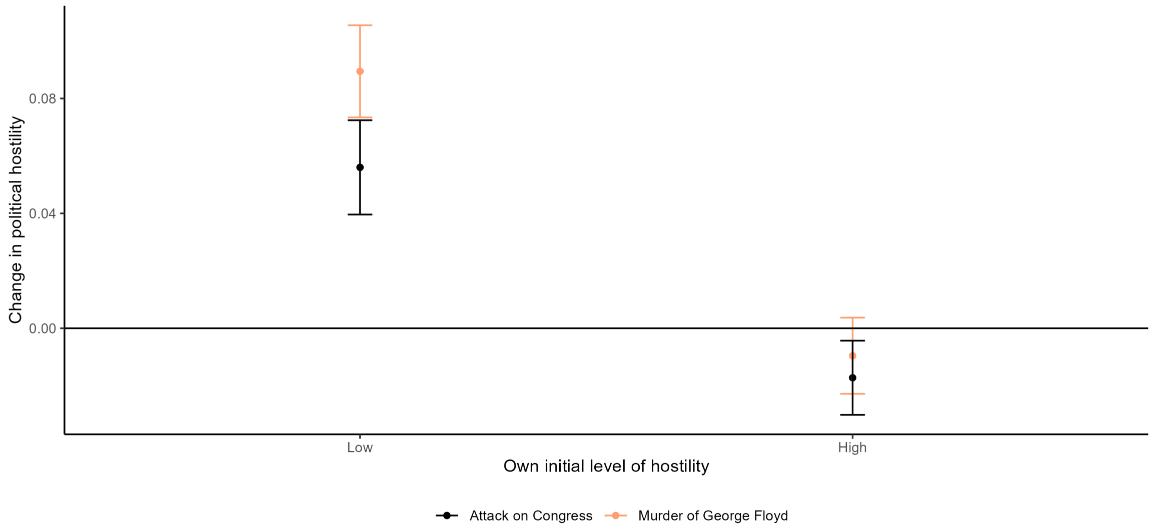

The figure illustrates the effect of the attack on Congress and the murder of George Floyd using a single time dummy for those initially low and high on political hostility

APSR Submission Template APSR Submission Template APSR Submission Template APSR Submission Template APSR Submission Template APSR Submission Template APSR Submission Template APSR Submission Template

FIGURE S15. The effect of the attack on Congress and the murder of George Floyd on number of tweets as a function of ideological diversity, political hostility in the network for those self-identifying as Republicans

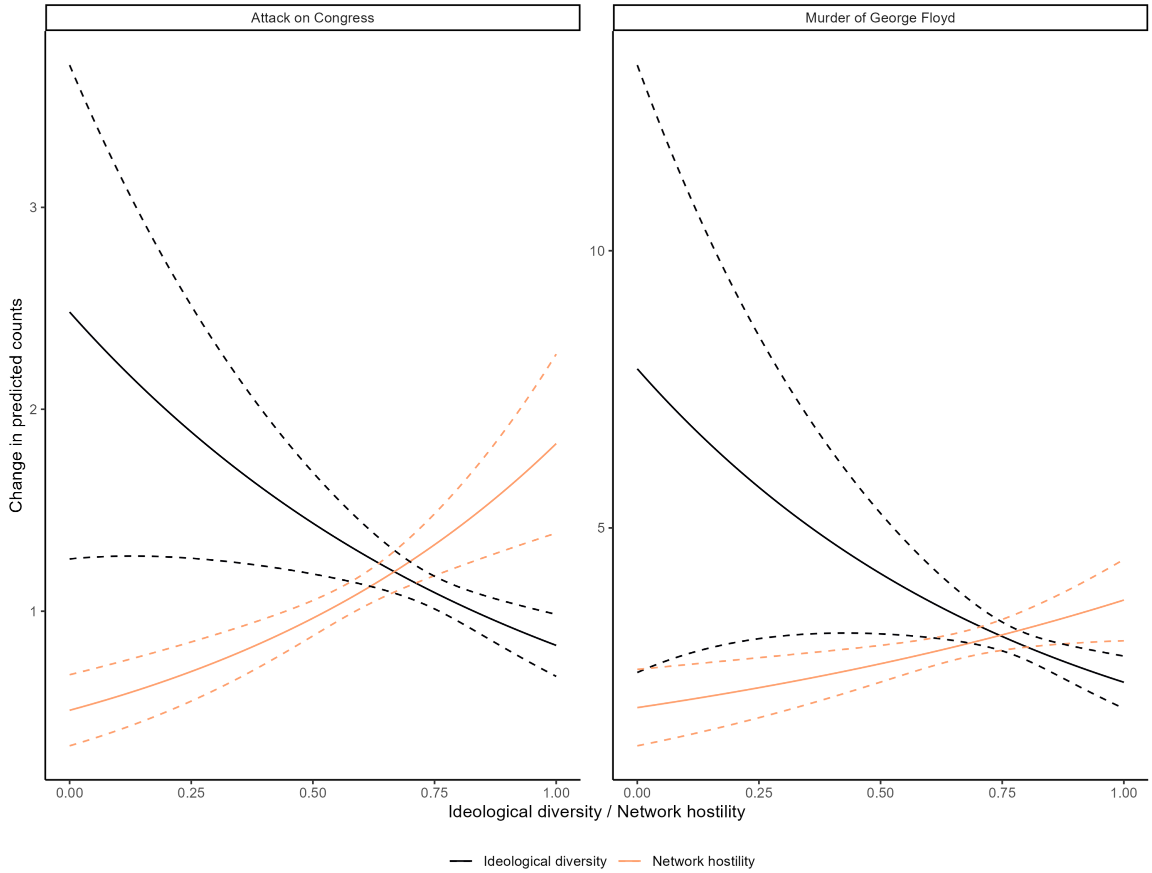

APSR Submission Template

FIGURE S16. The effect of the attack on Congress and the murder of George Floyd on number of tweets as a function of ideological diversity, political hostility in the network for those self-identifying as Democrats

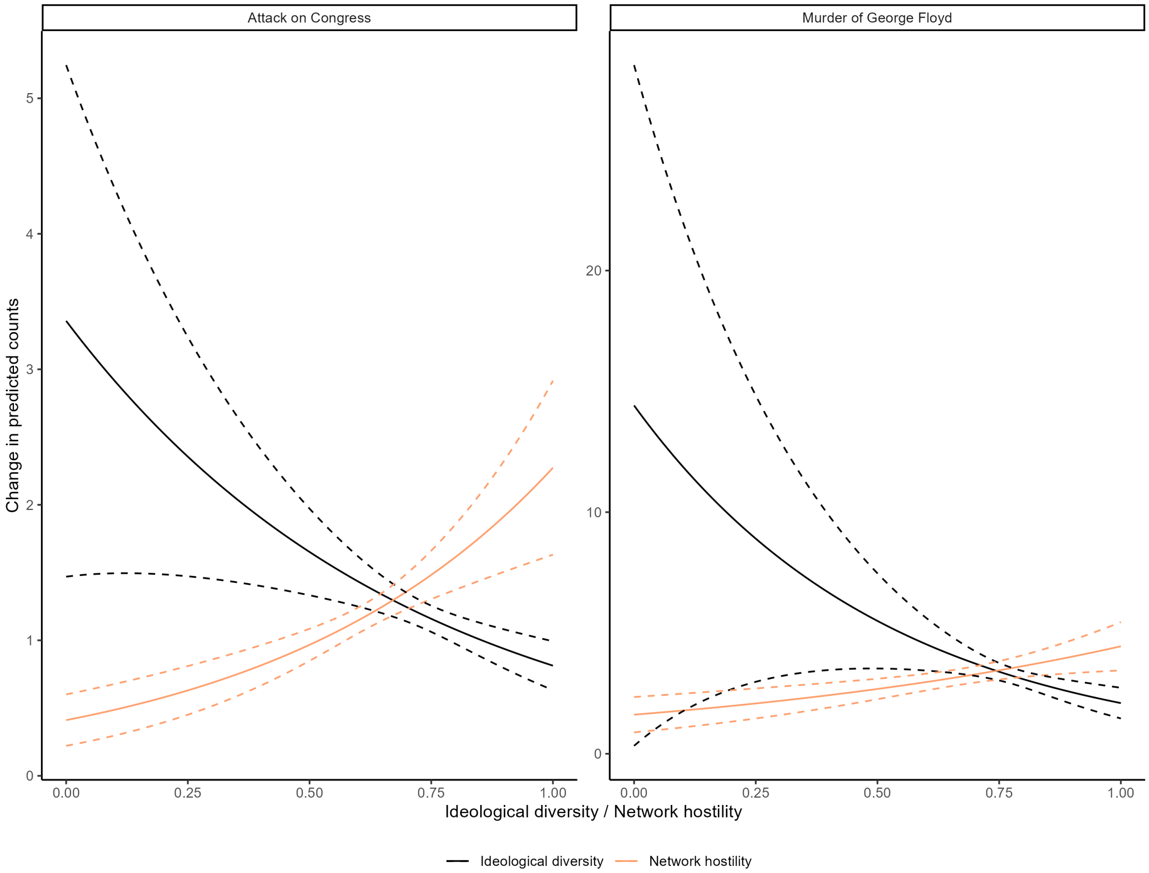

APSR Submission Template APSR Submission Template APSR Submission Template APSR Submission Template APSR Submission Template APSR Submission Template APSR Submission Template APSR Submission Template APSR Submission Template

FIGURE S17. The effect of the attack on Congress and the murder of George Floyd on political hostility as a function of hostility or ideological diversity in the network for those self-identifying as Republicans

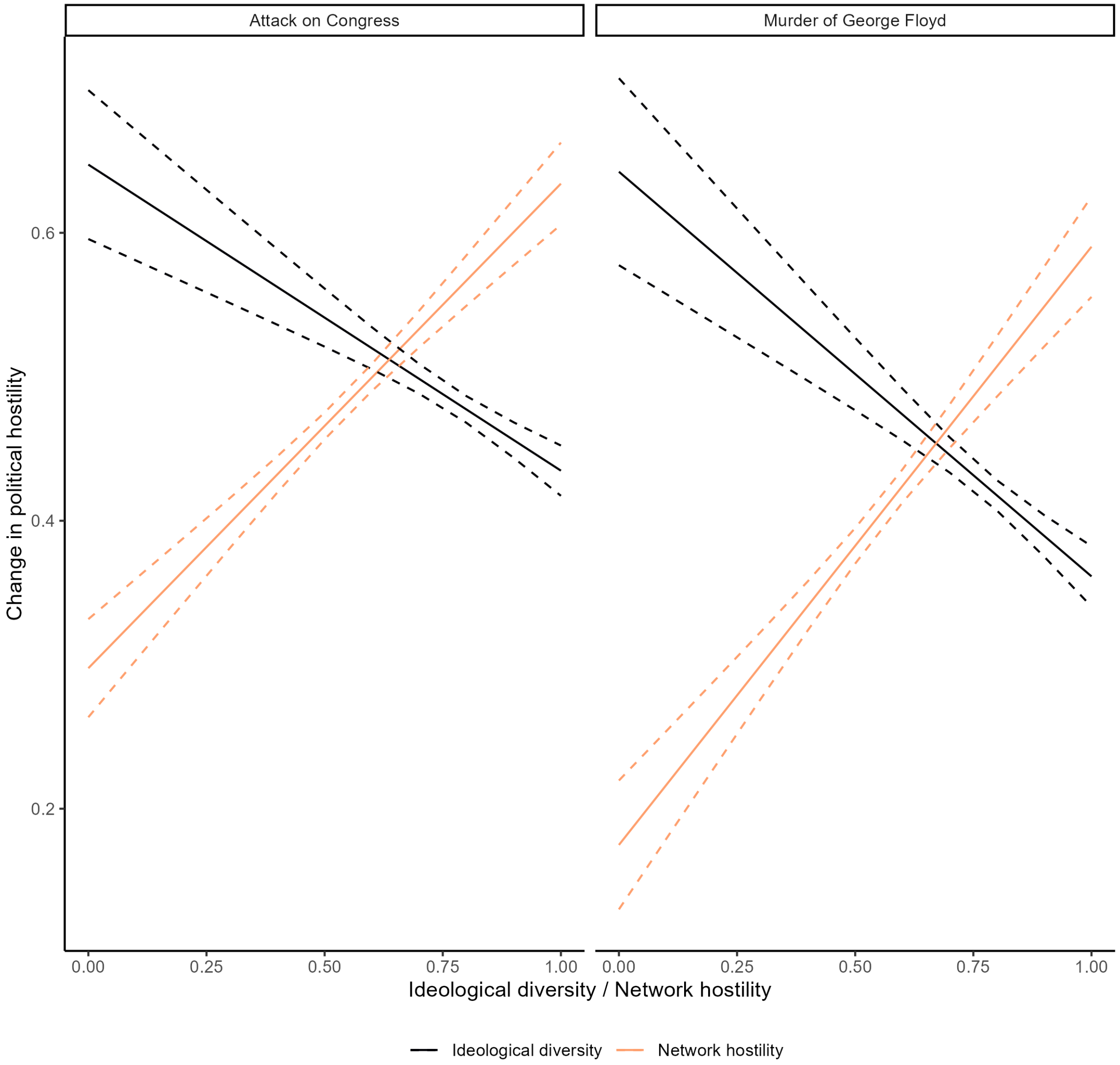

APSR Submission Template

FIGURE S18. The effect of the attack on Congress and the murder of George Floyd on political hostility as a function of hostility or ideological diversity in the network for those self-identifying as Democrats

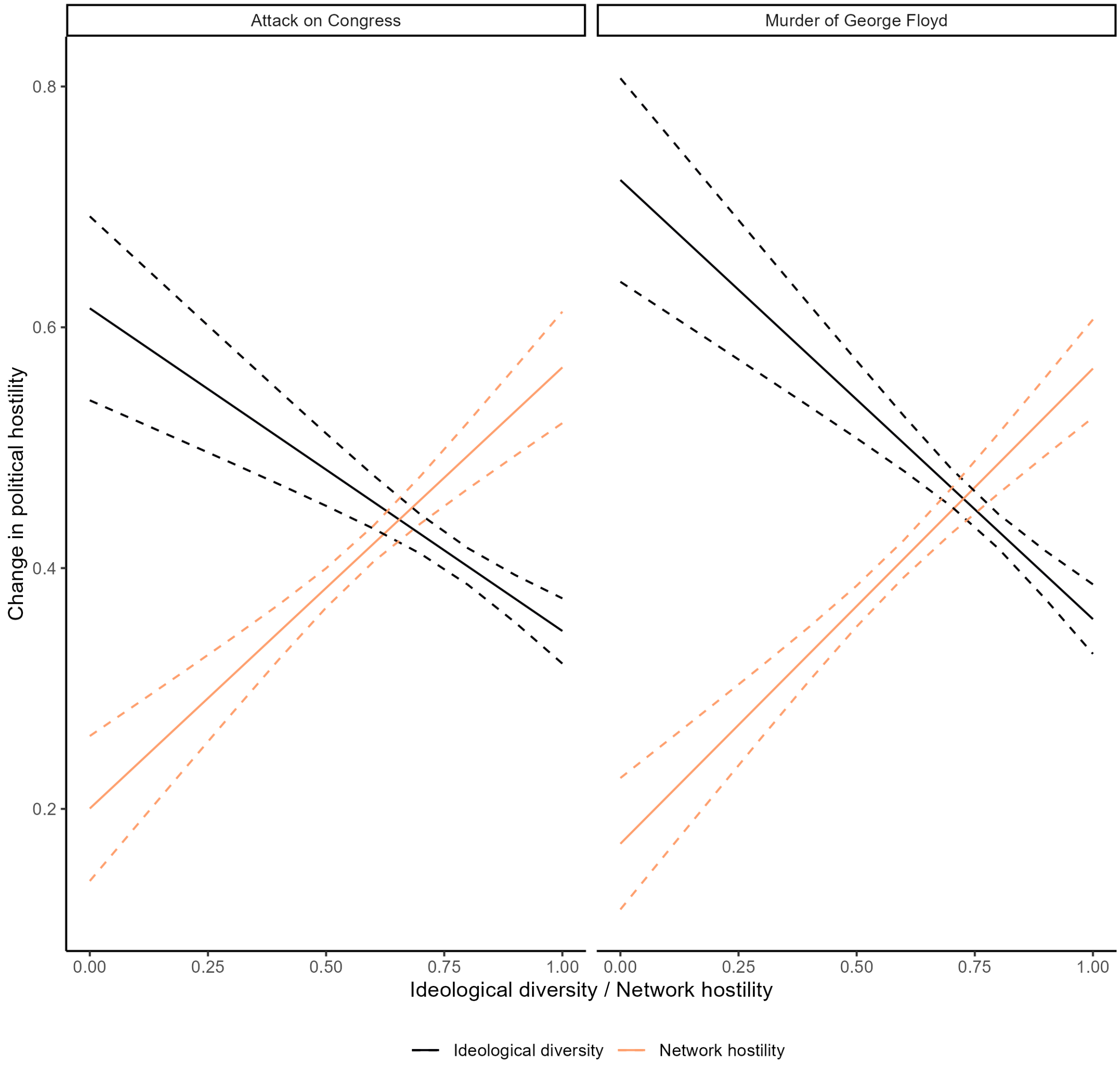

APSR Submission Template APSR Submission Template APSR Submission Template APSR Submission Template APSR Submission Template APSR Submission Template APSR Submission Template APSR Submission Template

APSR Submission Template APSR Submission Template APSR Submission Template APSR Submission Template APSR Submission Template APSR Submission Template APSR Submission Template APSR Submission Template APSR Submission Template

A2: Elaboration on BERT

BERT (Bidirectional Encoder Representations from Transformers), a state-of-the-art language model developed by Google, has revolutionized natural language processing tasks (Devlin et al., 2018). Although not the first model to use transformers it was one of the first large models to really demonstrate and apply this idea to a wide range of tasks for a big language model.

Transformers represent a groundbreaking development in the field of natural language processing and have revolutionized various language-related tasks (Vaswani et al., 2017). Unlike traditional models that process text sequentially, Transformers introduce the concept of self-attention mechanisms, enabling them to capture long-range dependencies and contextual relationships between words in a more efficient and effective manner. This attention mechanism allows Transformers to assign varying degrees of importance to different parts of the input sequence, enabling them to focus on the most relevant information. This parallel processing capability, coupled with the ability to capture both left and right context simultaneously, makes Transformers highly adept at understanding and generating language. Furthermore, Transformers have overcome the limitations of fixed-length context windows by introducing the concept of positional encoding, which enables them to retain positional information without relying on recurrence or convolution. This positional encoding, combined with the self-attention mechanism, empowers Transformers to capture contextual relationships across the entire input sequence. Additionally, Transformers can be pre-trained on large corpora, learning rich representations of language that can be fine-tuned for specific downstream tasks, resulting in remarkable performance gains. The combination of parallel processing, attention mechanisms, positional encoding, and pre-training/fine-tuning capabilities makes Transformers a breakthrough development in the field of natural language processing, enabling significant advancements in tasks such as machine translation, text generation, sentiment analysis, question answering, and more.

In the context of political online texts, BERT can be utilized to create a powerful classifier since it is already pre-trained on a huge amount of text data and therefore contains a lot of information about language in general. To begin using BERT, a dataset of political online texts needs to be collected, encompassing news articles, blog posts, social media content, and other relevant sources that cover a diverse range of political topics and viewpoints. Once collected, the data undergoes preprocessing

steps including the removal of irrelevant metadata, tokenization, and noise reduction. The next step involves fine-tuning BERT on the collected dataset by introducing a classification layer on top of the pre-trained model and training the network on the political text classification task.

The performance of the political text classifier is evaluated using separate test datasets and relevant metrics such as accuracy, precision, and recall. Cross-validation techniques like k-fold cross-validation can be used for more reliable performance estimates. Once trained and evaluated, the classifier can be deployed to classify new, unseen political texts. It can categorize texts into predefined classes, predict sentiment scores or political affiliations, and enable applications such as sentiment analysis, monitoring online political discourse, identifying misleading information, or personalizing political content based on user preferences. BERT provides a powerful framework for developing robust and accurate political text classifiers, contributing to a deeper understanding of political discourse, public opinion, and societal trends in the digital age.

Several papers in political science has already employed this method for social science constructs such as the study of online political hostility (Hebbelstrup Rye Rasmussen et al., 2023) and political sentiment in Germany (Widmann and Wich, 2022)

APSR Submission Template APSR Submission Template APSR Submission Template APSR Submission Template APSR Submission Template APSR Submission Template APSR Submission Template APSR Submission Template APSR Submission Template
